# Epidemiology of age-dependent prevalence of Bovine Herpes Virus Type 1 (BoHV-1) in dairy herds with and without vaccination

**DOI:** 10.1186/s13567-020-00842-5

**Published:** 2020-09-25

**Authors:** Jonas Brock, Martin Lange, Maria Guelbenzu-Gonzalo, Natascha Meunier, Ana Margarida Vaz, Jamie A. Tratalos, Peter Dittrich, Michael Gunn, Simon J. More, David Graham, Hans-Hermann Thulke

**Affiliations:** 1 Helmholtz Centre for Environmental Research GmbH-UFZ, Dept Ecological Modelling, PG Ecological Epidemiology, Leipzig, Germany; 2 Animal Health Ireland, Co. Leitrim, Carrick-on-Shannon, Ireland; 3 Centre for Veterinary Epidemiology and Risk Analysis, UCD School of Veterinary Medicine, University College Dublin, Dublin, Ireland; 4 Faculty of Veterinary Medicine, University of Lisbon, Lisbon, Portugal; 5 Department of Mathematics and Computer Sciences, Friedrich Schiller University, 07743 Jena, Germany

**Keywords:** infectious Bovine Rhinotracheitis (IBR), infection dynamics, Ireland, seasonal calving, serosurveillance, dairy

## Abstract

Many studies report age as a risk factor for BoHV-1 infection or seropositivity. However, it is unclear whether this pattern reflects true epidemiological causation or is a consequence of study design and other issues. Here, we seek to understand the age-related dynamics of BoHV-1 seroprevalence in seasonal calving Irish dairy herds and provide decision support for the design and implementation of effective BoHV-1 testing strategies. We analysed seroprevalence data from dairy herds taken during two Irish seroprevalence surveys conducted between 2010 and 2017. Age-dependent seroprevalence profiles were constructed for herds that were seropositive and unvaccinated. Some of these profiles revealed a sudden increase in seroprevalence between adjacent age-cohorts, from absent or low to close to 100% of seropositive animals. By coupling the outcome of our data analysis with simulation output of an individual-based model at the herd scale, we have shown that these sudden increases are related to extensive virus circulation within a herd for a limited time, which may then subsequently remain latent over the following years. BoHV-1 outbreaks in dairy cattle herds affect animals independent of age and lead to almost 100% seroconversion in all age groups, or at least in all animals within a single epidemiological unit. In the absence of circulating infection, there is a year-on-year increase in the age-cohort at which seroprevalence changes from low to high. The findings of this study inform recommendations regarding testing regimes in the context of contingency planning or an eradication programme in seasonal calving dairy herds.

## Introduction

Bovine Herpes Virus Type 1 (BoHV-1) is a highly contagious virus of cattle and is known to be the causative agent of the acute respiratory disease infectious bovine rhinotracheitis (IBR). The virus occurs worldwide and is endemic in a number of food-producing countries [1]. Following infection, cattle are lifelong carriers of the virus with the potential of spontaneously reactivated viral shedding, especially at times of increased stress [2]. Infections with BoHV-1 may be associated with various clinical signs and changes in production parameters, ranging from fever, reduced growth rate and milk yield to an increased risk of abortion and death [3, 4].

When a naïve animal is exposed to BoHV-1, a so called primary infection can occur [2] and virus is excreted in nasal fluid over a period of 10–17 days with a peak at 4–6 days post‐infection [5]. During the course of a primary infection, animals shed high levels of virus (up to 3800 000 TCID_50_/mL nasal discharge; [6, 7]) and are considered the main spreaders of infection during an outbreak. In experimental studies, the number of secondary cases generated by a single primary infected animal, the basic reproduction number (R0), is estimated between 3.2 and 7 in herds with susceptible animals [8–11]. Following infection, cattle become latently infected. At this stage of infection, animals are virus carriers, seropositive, but neither shed infectious material nor exhibit clinical signs. Usually, latently infected animals are identified by the detection of BHV-1-specific antibodies in their serum. Studies have shown that antibodies persist at stable levels in experimentally infected cattle for at least 2–3 years [12]. BoHV-1 shedding can reactivate within latently infected animals (secondary infection), resulting in viral transmission and the establishment of primary infections in other naïve cattle [2].

A number of alternative pathways for BoHV-1 transmission have been reported in the literature. Direct contact between animals (e.g. nose to nose) is considered the most important route of transmission within farms [2]. Aerosol transmission can occur over short distances of up to five metres, and as such may result in between-herd transmission across farm boundaries [13]. However, the purchase of latently infected animals is considered to be the main source of between-herd spread.

Infections with BoHV-1 occur worldwide, although there are differences in prevalence and incidence [1]. In the European Union (EU), a number of countries or regions are considered free from BoHV-1 following the implementation of EU-approved eradication programmes [14], including Austria, Germany, Denmark, Finland, Sweden, Jersey (United Kingdom) and Valle d’Aosta and the Province of Bolzano (Italy) [15] and as such are granted additional guarantees in respect of trade to protect this status under Article 10 of Directive 64/432/EEC. Switzerland and Norway are also considered free of infection, although they do not have formal Article 10 status as non-EU members. Other countries and regions within the EU are currently implementing Commission-approved eradication programmes, including Belgium, Czech Republic, Luxembourg and the province Friuli-Venezia Giulia in Italy [15] and are again granted additional guarantees in terms of trade under Article 9 of Directive 64/432/EEC. In both cases, these additional guarantees impact on the trade of live cattle from countries or regions not free from BoHV-1, particularly when they do not have approved eradication programmes.

In the Republic of Ireland, information on BoHV-1 prevalence is available from recent cross-sectional studies [16–19]. These studies indicate that BoHV-1 is endemic, with 75–90% of Irish herds positive for BoHV-1 antibodies. Vaccination has been introduced to reduce herd prevalence. However, the legal use of non-marker vaccines ceased at the end of 2004. Since then, the only type of vaccine licensed in the Republic of Ireland are marker vaccines, allowing differentiation between field virus exposure and vaccination. A national programme to control BoHV-1 does not currently exist in Ireland, but is under active discussion, co-ordinated by Animal Health Ireland (AHI; www.animalhealthireland.ie). The current study was undertaken to inform the consideration of options for a national eradication programme in Ireland.

An understanding of the dynamics of BoHV-1 seroprevalence within herds is essential to guide control decisions and inform programme design. The published literature contains a number of retrospective studies where age is repeatedly identified as a risk factor for BoHV-1 seropositivity [e.g. 10, 17, 19–22]. It is unclear whether this pattern reflects true epidemiological causation or is a consequence of study design. Moreover, an improved understanding of the dynamics of serological patterns in BoHV-1 positive herds is important as a basis for evaluating the effect of vaccination on within-herd seroprevalence.

The objectives of this study were (1) to understand the age-related dynamics of BoHV-1 seroprevalence in unvaccinated dairy herds with seasonal calving, and (2) to determine how vaccine usage affects these dynamic patterns. The findings are discussed in the context of IBR control and surveillance.

## Materials and methods

### BoHV-1 data

The data used for this study originated from two Irish seroprevalence studies undertaken by the Moorepark Animal and Grassland Research and Innovation Centre, managed by Teagasc (The Agriculture and Food Development Authority), between the years 2010 and 2017. The data have already been used in previous studies and we refer to [23] for details of these BoHV-1 serological surveys.

In brief, the first seroprevalence study was carried out on 24 dairy herds from which blood samples were taken at least once between 2010 and 2013. Herds selected for the study were Teagasc research farms or participants in the Dairy Information System [24]. All antibody tests were performed using IDEXX ELISA test kits appropriate to the vaccination status of the herd, i.e. using gE or gB tests. Antibody tests were classified as positive, negative or inconclusive based on the sample-to-positive ratio using cut-off thresholds in accordance with the manufacturer’s guidelines [23]. These data were supplemented by the results of additional bleeds of a subset of herds between 2015 and 2017. Information on the vaccination status of each herd was also collected, i.e. whether the herd was vaccinated or not, type of vaccine used and frequency of vaccination.

The second seroprevalence study was conducted in 2015 on 57 dairy herds. Herds under investigation were members of a breeding information service (HerdPlus; https://www.icbf.com/wp/?page_id=149), provided by the Irish Cattle Breeding Federation (ICBF). Blood samples were collected from the entire herd, tested for BoHV-1 antibodies and the herd´s vaccination status recorded. Information on the frequency of vaccination and the type of vaccine used were not recorded. Antibodies were determined using IDVet gE and Qiagen gB ELISA in vaccinated and non-vaccinated herds, respectively. Again, the sample-to-positive ratio was used for classifying antibody titres as positive, negative or inconclusive by referring to the manufacturer’s instructions.

Based on information provided by the manufacturers, the test parameters for serological diagnosis ranged between 0.974 and 1 for sensitivity and 0.996 and 1 for specificity.

Merging both datasets resulted in 81 surveyed dairy herds with 23,459 individual BoHV-1 tests results.

### Data processing & extraction of datasets

Data were managed in a Microsoft Access database and screened for errors and missing data. Analysis and visualization was conducted entirely in R [25]. All animals with an inconclusive BoHV-1 antibody test result (n = 89) were removed from further analysis. Based on the herd number, movement data were extracted from the Animal Identification and Movement (AIM) database maintained by the national Department of Agriculture, Food and the Marine (DAFM). From January 2009 until December 2017, all in- and outward cattle movements per herd were matched with individual serology test data.

Herds were excluded from the dataset if the number of animals introduced each year was higher than 5% of the herd’s average size or the yearly out-moves exceeded 50%. This is critical when analysing the dynamics of the within-herd seroprevalence (percentage of seropositive cattle in a herd) to ensure that potential increases/decreases in prevalence are not dominated by moves into or out of each herd.

Based on the date of birth of each tested animal, we calculated the age in days at the time of sampling. Animals were then grouped into age cohorts by year from 0 to 9. Animals older than 9 years were assigned to cohort > 9.

BoHV-1 seroprevalence was estimated at herd and age-cohort level for each round of sampling, calculated as the number of seropositive cattle divided by the total number of cattle tested. For each age cohort, a Gaussian confidence interval was calculated on the basis of the apparent prevalence of seropositive animals at the 95% significance level.

In a final step, we created two overlapping subsets of the data (A & B). Dataset A included serological results from herds that at the time of sampling were not vaccinated against BoHV-1. All herds that were seronegative or not completely sampled (e.g. missing young-stock data) were excluded. For herds that were tested multiple times the data of the first sampling was used. In dataset A, each herd was included only once. Thus 3994 test results from 15 unvaccinated seropositive dairy herds were used for dataset A. Dataset B included all herds that were tested at least twice, irrespective of their vaccination status. All test results from these herds were included in dataset B, with the exception of results from herds that were not completely sampled for a given round of testing. 8972 test results from 13 multiple-tested herds were retained in dataset B. These multiple tested herds were investigated to study the influence of vaccination on the within-herd prevalence. Six herds were already vaccinating before sampling was conducted or had started to vaccinate within the sampling period. All herds in dataset B showed a decline in herd-level/age-related seroprevalence over time (see Additional file 3).

### Modelling the spread of BoHV-1 in dairy cattle

#### Model description

A stochastic, individual-based, herd-level simulation model of BoHV-1 infection was developed to demonstrate the dynamics of age-dependent patterns of seroprevalence in BoHV-1 seropositive herds and provide a qualitative comparison with those of our serology data (Dataset A). The documentation of the model follows the ODD protocol (overview, design concepts, and details); [26–28] and is provided in the supplementary material (see Additional file 1).

The model simulates the spread of BoHV-1 in a seasonal calving dairy herd, where young stock and lactating cows are not epidemiologically separated and replacement stock are home-bred. The simulated herds are similar in structure (e.g. 120 breeding females) and management practices. The herds are simulated without in-moves and hence represent epidemiologically closed herds. The model simulations are not intended to replicate the herds in the data but rather to highlight general BoHV-1 serology dynamics. The model design comprises two components [29]. The demographic component captures relevant biological and farming-related processes, and the pathogen-related component describes the transmission and spread of BoHV-1 after the virus has been introduced. The simulation model runs in weekly time-steps and is implemented in Scala (https://www.scala-lang.org/).

#### Modelled scenarios

We simulated 15 separate dairy herds, each for 20 years. BoHV-1 infection was seeded in all herds at the same point in time by infecting a random animal (seed infection).

In Scenario A, we simulated the course of infection in the herds while excluding reactivation of latently infected cattle. The serological status of each animal in the simulated herd was recorded during the simulation period. The seroprevalence profiles across age-cohorts of the simulated herd were evaluated at three intervals: immediately after infection (6 months after the initial seeding of infection); at a time point selected at random between years two and five, and at 12 years after the initial seeding of infection.

Scenario B was similar to A but this time a reactivation event was simulated 4.5 years after the initial seeding of infection. The reactivated individual was forced to infect one naïve animal, thereby triggering a primary infection during its infectious period. Again, three seroprevalence profiles were reported: at 6 months, at 5 years (i.e. 6 months after the reactivation event), and at a time point selected at random between nine and 12 years after the initial seeding of infection (i.e. 4.5 to 7.5 years after the reactivation event).

## Results

### Within-herd seroprevalence in unvaccinated dairy herds

Among the 17 unvaccinated dairy herds, two herds returned 100% negative test results. 15 dairy herds were non-negative (Dataset A) and within-herd seroprevalence varied between 0.8 and 96.2%. The overall seroprevalence in these 15 positive herds was 31.7% (SD 8%).

**Figure 1:**
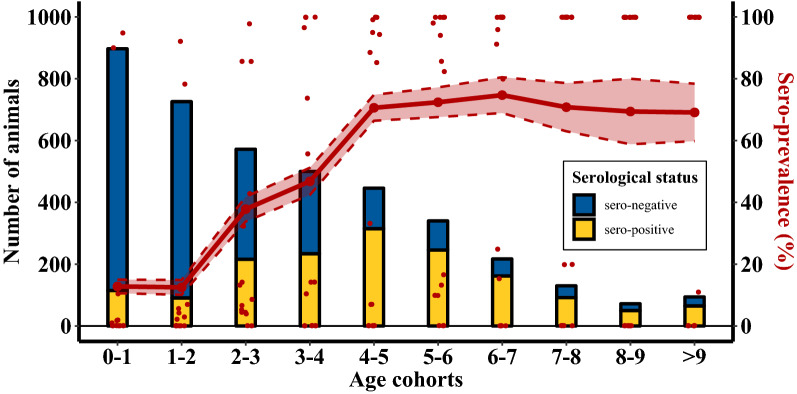
**Animal-level seroprevalence in 15 unvaccinated seropositive dairy herds (Dataset A), by age cohort.** Per age cohort the dataset is shown as the absolute number of seropositive/negative cattle (yellow/blue bar chart, left y-axis); and as a proportion of seropositive animals (red line, right axis, with its 95% confidence interval). Red dots (right axis) refer to herd-level seroprevalence for each herd, again by age-cohort. (Note: Across the early and late age-cohorts the individual herds’ prevalence values (red dots) tend to dichotomies between zero and 100%).

The overall seroprevalence of antibodies against BoHV-1 in these herds increased with age (Figure 1). The overall proportion of seropositive animals was low (< 20%) in younger animals (0–2 years), increasing in cattle aged between two and five years old (from approximately 20% to 70%). In all older cohorts, the overall proportion of seropositive animals was approximately 70%.

**Figure 2:**
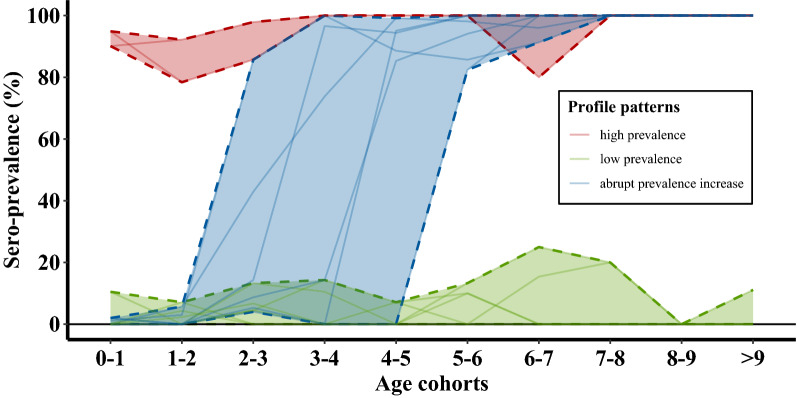
**Individual age-dependent seroprevalence profiles of 15 unvaccinated seropositive dairy herds (Dataset A as in Figure** **1****).** Each line represents the age-dependent data from a single herd. Three qualitatively different profile categories are identified: [1] herds with a high seroprevalence throughout all age cohorts (red), [2] herds with one abrupt increase in seroprevalence between consecutive age cohorts (blue), and [3] herds with a low prevalence across all age cohorts. Solid lines represent individual herds, while dashed lines indicate maximum/minimum envelopes per category.

Figure 2 reassembles the data used in Figure 1, however, instead of summarizing data across the herds, the age-dependent seroprevalence profiles were plotted separately for each herd. The individual age-dependent seroprevalence profiles differed from each other. Three main patterns were observed, which suggests three different herd categories. The first category includes herds showing a very high prevalence throughout all age cohorts (red lines; two herds out of 15). In these herds, an increase in seroprevalence with age was not obvious. The second category of herd profiles has very low seroprevalence across all age cohorts (green lines; seven herds out of 15); again, no increase by age-cohort was seen. The last category of prevalence profiles includes those herds in which seroprevalence changes abruptly from low to high, typically between consecutive age cohorts (blue lines; six herds out of 15). The age cohorts in which this step-like increase occurred was variable, here ranging from 2–3 to 5–6 years.

**Figure 3:**
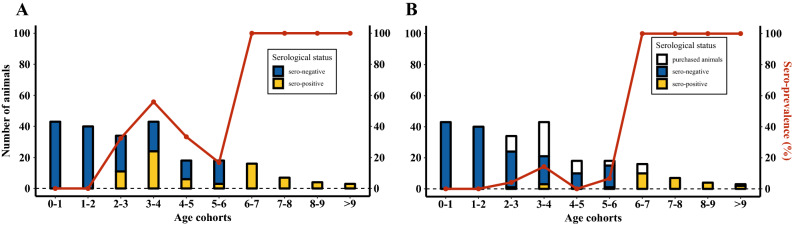
**Serological profile of a single unvaccinated herd before (A) and after (B) exclusion of purchased animals.** Correction for movement events reveals the category of seroprevalence profiles reflecting the history of BoHV-1 circulation in this herd. Note: Nevertheless, this herd was excluded from further analysis due to the high number of in-moves.

Outputs presented in Figures 1 and 2 show patterns of age-dependent seroprevalence profiles if trade of animals was excluded. In practice many herds will not be closed (i.e. through homebred replacement) with movements both into and out of the herd over several age-cohorts. As a consequence, the age-dependent seroprevalence profiles may become noisier. Figure 3 presents an example herd and provides a picture of how in-moves can mask the critical step-like increase in the age-dependent seroprevalence profile of the herd. This example herd was initially excluded from further analysis due to the large number of in-moves into this herd. Without the exclusion of purchased animals, almost all of which were younger than 6–7 years of age, the steep increase in seroprevalence between consecutive age-cohorts at 6–7 years was not evident (Figure 3A). Exclusion of introduced animals from the calculation of the age-dependent seroprevalence profile makes the critical age-related threshold visible (Figure 3B).

### Modelling BoHV-1 within-herd spread

**Figure 4:**
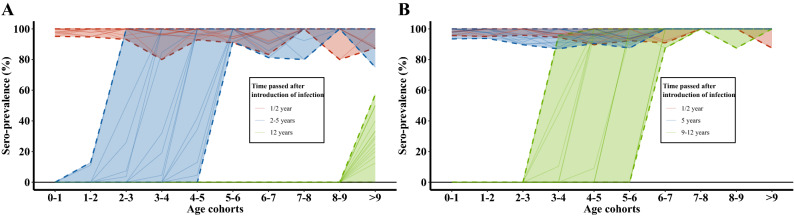
**Model output of the age-dependent seroprevalence profiles of 15 simulated cattle herds at three subsequent time points after a single introduction of BoHV-1 infection (line colours).** Solid lines represent individual herds, while dashed lines indicate the maximum/minimum envelopes per time point. **A** Output scenario A—no reactivation: the 15 seroprevalence profiles are reported at 0.5 years (red); at a random time point between 2 and 5 years (blue); and at 12 years (green) after the initial seeding of infection. **B** Output scenario B—reactivation of a latent animal was forced 4.5 years after the initial seeding of infection, leading to an additional round of primary infection: the 15 seroprevalence profiles are reported at 0.5 years (red); at 5 years (blue); and at a random time point between 9 and 12 years (green) after the initial seeding of infection. Note: In (b) Blue lines, i.e. 5 years post introduction, equal 0.5 years after reactivation occurred; and green equals time points selected at random between 4.5 and 7.5 years after the reactivation event.

Results from the individual herd-based simulation model are presented in Figure 4. With scenario A (no reactivation; Figure 4A), the simulated age-dependent seroprevalence profiles show similar patterns to those observed in the field data. No age-dependent trend is apparent in the seroprevalence profiles of the 15 individual herds 6 months after the initial seeding of infection (red lines). In the simulated herds, nearly all stock had seroconverted within half a year. A step change in seroprevalence from low to high between consecutive age-cohorts is observed in the subsequent seroprevalence profiles at time points scheduled at random between two and 5 years after the initial seeding of infection (blue lines). In this modelling scenario, reactivation was completely suppressed; therefore circulation of virus will reduce over time and animals born later (i.e. younger age-cohorts) are not exposed to infection. The last seroprevalence profiles are taken 12 years after the initial seeding of infection (green line). These profiles were nearly free of BoHV-1 seropositive animals due to the ongoing replacement of animals during normal management processes. Further, viral fade-out has already removed the majority of seropositive cattle that were infected during the original period of virus circulation. Only animals surviving into the oldest age-cohort (> 9y) remain in this profile.

In scenario B (Figure 4B), a reactivation event was triggered four and half years after initial virus introduction, leading to new primary infections in naïve animals. At the point when reactivation occurred, a proportion of seropositive animals, i.e. older animals which had seroconverted following the first period of extensive virus circulation 5 years previously, were still present in the simulated herds. However, the new primary infections caused another major episode of virus circulation and hence seropositivity was again high in all age-cohorts at the time of testing 6 months after reactivation (blue lines; 5 years after the initial introduction of infection). Comparing the individual age-dependent seroprevalence profiles (red vs. blue), it is not possible to distinguish age-related serological outcome between “subsequent” outbreaks induced by reactivation (blue lines) and “initial” outbreaks due to introduction into a naïve herd (red lines). Moreover, it is not possible to distinguish age-dependent serology profiles more than 5 years after reactivation (Figure 4B; green lines) with those up to 5 years after naïve introduction (Figure 4A; blue lines). Therefore, at the level of age-dependent seroprevalence profiles in individual herds, any new period of virus circulation effectively acts like a resetting of the clock. The key information provided by analysis of these herd profiles is the period of time during which the herd was free of circulating infection, as indicated by the absence of new seroconversions. This time interval is equivalent to the age-cohort in which the step increases occur, as shown in Figure 2.

### Effect of vaccination on the within-herd prevalence

**Figure 5:**
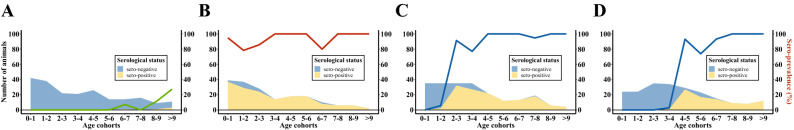
**Stacked area graphs representing the age-dependent seroprevalence profile over a five year period in a single vaccinated dairy herd.** The herd was serologically sampled in (**A**) 2010, (**B**) 2011, (**C**) 2013 and (**D**) 2015. In this herd, vaccination started in 2012. The left y-axis (the number of animals) refers to the smoothed histograms (seropositive, seronegative) and the right y-axis (seroprevalence) to the coloured line. The lines represent age-dependent seroprevalence profiles, which were coloured according to the epidemiological stages as outlined in Figure 2.

With the intention of understanding how vaccination might affect herd-level seroprevalence, we selected a single case herd from dataset B and analysed the change of age-dependent prevalence under vaccination over time (Figure 5). This herd was selected as it had the largest number of sample points. From the beginning of 2009 until the end of 2017, this herd did not purchase any cattle and can thus be considered as closed. In 2010 (Figure. 5A), the selected herd was non-vaccinating and was seronegative except for a few individuals in older age cohorts. One year later, in 2011 (Figure 5B) almost every animal in the herd had seroconverted as a result of active virus circulation. A vaccination programme was initiated in 2012 and it is evident from the data that no new virus circulation occurred in the herd since then (Figure 5C and D). Replacement of older seropositive stock by seronegative home-bred young-stock led to the expected shift in the age-dependent seroprevalence profile. In Figure 5D the critical jump occurred between age-cohorts 3–4 and 4–5 (born in the spring of 2012 and 2011 respectively). Hence, the stock born during these three “preceding” years, beginning in 2012 and corresponding to the 3 years since vaccination was introduced remained free of infection, i.e. absence of spread of infection. Consistent with this, the most recent seroconversions in Figure 5D were found in age-cohort 4–5 i.e. the animals from age-cohort 0–1 in 2011 when the major outbreak had occurred (5b). A similar pattern was also evident in the other vaccinating herds with minimal purchases (see Additional file 3).

## Discussion

### Age-dependent patterns of BoHV-1

Understanding the age-dependent patterns of BoHV-1 spread is essential to accurately interpret trends in serological data and to inform decision-making in the context of the design and evaluation of control strategies such as vaccination. To our knowledge, this work is the first to explicitly explore the relationship between age-related serological profiles in seasonal calving dairy herds and BoHV-1 infection dynamics. The purpose of doing so was to improve the interpretation of surveillance outputs based on seroprevalence figures.

In this study we confirmed that, in herds with positive serological profiles, older cattle most commonly have antibodies against BoHV-1 (Figure 1). This is consistent with other studies [e.g. 10, 17, 19–22], and would seem to concur with earlier suggestions of increased infection risk with age, or with age as a proxy measure of exposure time in combination with lifelong seropositivity. However, we caution against this conclusion. Indeed, the inspection of individual herd-level seroprevalence profiles highlights the pitfall of this interpretation which is derived from analyses conducted on aggregated data. As demonstrated in Figure 2, there are no examples in any of the 15 unvaccinated dairy herds of a smooth increase in seroprevalence over several age-cohorts to saturation level. In fact there are only two distinct situations of equivalent levels of seroprevalence (either low (< 20%) or high (> 80%)) across all age-cohorts. These findings are consistent with the literature; if BoHV-1 is actively circulating within a naïve herd, most animals will seroconvert within a short period of time [e.g. 9, 10, 29, 30]. These major outbreaks are a result of the high level of infectiousness of the virus. Different field studies have estimated the reproduction number (R0) of the infection between 3.2 and 7 in unvaccinated herds [8–11].

The sudden increase in seroprevalence between consecutive age-cohorts that is observed in the individual herd-level profiles (Figure 2) reflects extensive viral circulation within a herd for a limited time, which may then remain latent over the following years [10]. Several studies report extended periods of time with no evidence of BoHV-1 circulation in endemically infected herds [31, 32]. Following a fade-out in virus circulation, subsequent (i.e. younger) age-cohorts in the herd can only seroconvert following either introduction of new, actively infected animals into the herd or reactivation of latently infected animals leading to new primary infections. In our data, termination of virus circulation was evident, namely in the herds characterized by the abrupt increases in the seroprevalence profiles (Figure 2, blue lines). In these herds, virus circulation stopped between three and 5 years before serological sampling i.e. the age cohort where the abrupt increase in seroprevalence occurred. The observed pattern is particularly evident in seasonal-calving dairy herds in Ireland, where the (home-bred) population increases in a step-wise fashion and not continually over the year. However, model simulations relaxing the assumptions of seasonal calving did reveal the same feature of a sudden increase stretched over more than one age-cohort due to the continued replacement (see Additional file 2).

Bull breeding might cause a potential seroprevalence jump at age cohort 1–2 i.e. first breeding of naive heifers [33]. And, by every years breeding season the infected bull potentially might cause successive age-cohorts with high seroprevalence. However, in our data set this is not the case because (1) not all “jumps” occur at age 1–2 so respiratory spread must have been involved, (2) infection by bull would either create single age cohorts with high sero-prevalence in those years when naive heifers got infected or subsequent reactivations may have caused respiratory spread affecting all cattle on herd that time, (3) artificial insemination (AI) has become the breeding norm on the majority of dairy farms in the Republic of Ireland.

The pitfalls of aggregated analysis (Figure 1) are related to the fact that herds usually are serologically sampled at different epidemiological stages relative to the time when virus was actively circulating (Figure 2). Aggregated herd sampling, essentially an averaging across age-cohorts, leads to a blurring of the temporal stage and a misleading interpretation of the age-related seroprevalence data (Figure 1). It is therefore not surprising that, in some studies where aggregated data are used, older animals are reported as being at higher risk of BoHV-1 infection due to time in the herd [21].

The three categories of herd-based seroprevalence profiles, as presented in Figure 2, were derived post hoc based on patterns observed in the data. The simulation modelling revealed that the course of a BoHV-1 outbreak in a naïve herd, together with different sampling times during that outbreak history, mirror the categories identified from the data (Figure 4A). Prior to testing of herds, their temporal stage relative to fade-off in infection circulation (or introduction) is usually unknown. Therefore, for BoHV-1 survey data as described here, the number of herds in each of the three categories as well as the actual age-cohort with sudden increase of seroprevalence are a chance outcome. A summary of age-related seroprevalence across herds likely will, as an artefact, show the smooth increase of seroprevalence with age, as illustrated in Figure 1. This issue could be tackled by adjusting the individual seroprevalence profiles according to the age-cohort in which infection faded out, i.e. where the jump occurs between consecutive age-cohorts.

Finally, we need to understand whether reactivation may lead to a pattern of gradual increase in age-related seroprevalence profiles similar to that presented in Figure 1, i.e. we may speculate that a low reactivation rate in combination with a low infectiousness of animals following reactivation of latent infection is leading to limited exposure in younger age-cohorts and hence lower seroprevalence estimates in these age-cohorts. To the authors’ knowledge, the rate at which reactivation occurs in latently infected animals has not yet been determined experimentally. However, previous modelling studies assumed that the rate at which the virus is reactivated in latently infected animals lies between 0.13 and 2.6% per year [34–36]. Following reactivation of latent infection, animals have been shown in experimental studies to shed on average 6000 times less infectious virus than animals undergoing primary infection [6, 37]. Hence, the infection risk posed following reactivation of latent infection is much lower than with primary infection. Despite this, the literature includes studies where reactivation of latent infection in single animals induced major virus outbreaks [8]. This is plausible given that reactivation can result in ‘additional seroconversions’ if new primary infections are triggered, e.g. in the younger age-cohorts.

In our individual-based model (Figure 4B), the synthetic reactivation event did result in a primary infection and always triggered substantial viral circulation, even if the herd already had a substantial proportion of seropositive animals, i.e. in the older cohorts. Hence, the scenario whereby reactivation establishes a primary infection cannot result in a gradual increase in serological profiles by age. Therefore, age should not be reported as a risk factor in relation to IBR or BoHV-1 infection. Nevertheless, a randomly selected older animal is more likely to be tested seropositive for BoHV-1 antibodies than a randomly selected young animal. On average, when considering aggregated data across multiple herds, these older animals have a greater likelihood of having been involved in a past major outbreak.

**Figure 6:**
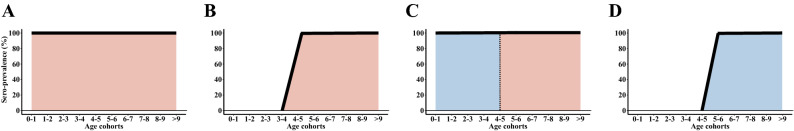
**Conceptual model for the spread and temporal development of BoHV-1 seroconversions in an unvaccinated dairy herd over the course of 10** **years.** A successful reactivation event occurred 5 years after initial virus introduction. In each of the plots, the black line represents apparent seroprevalence of a theoretical herd whereas pink represents seroconversions attributable to the initial outbreak and blue to the subsequent reactivation event. **A** Age-dependent seroprevalence profile half a year after initial virus introduction; **B** seroprevalence profile 4.5 years after virus introduction; **C** seroprevalence profile 5.5 years after virus introduction i.e. half year after reactivation event. **D** Seroprevalence profile 10 years after initial virus introduction and 5 years after the reactivation event.

Figure 6 is a theoretical representation of Figure 4B, with serological markers presented as either pink (as a result of the first major outbreak following virus introduction into the herd) or blue (as a result of the subsequent reactivation event). That is, the colours assist in visualising whether the serological status of the animals is a result of the initial introduction or a later reactivation. Virus reactivation is an important component of BoHV-1 dynamics, not because of the infectivity of animals undergoing secondary infection, but rather because of the potential for these animals to initiate further primary infections. Thus, it is reasonable when literature reports outbreaks induced by reactivation of the virus as indistinguishable from outbreaks due to new introduction of the virus from outside the herd [8].

The conceptualisation shown in Figure 6 suggests perfect ‘on–off’ dynamics of virus circulation. Although this is generally in agreement with field data as presented in Figure 2, several herd profiles incorporate noisy deviations from the conceptually expected 0% and 100% levels. There are a number of reasons why this might be the case. These include imperfect test characteristics of the ELISA or animal movements [9] highlight that with large transmission values of BoHV-1 (R0 = 7), theoretically up to 15% of the virus introductions may result in a small outbreak (i.e. the first primary infected animals by chance do not affect greater parts of the herd before virus circulation fades off), hence, leaving noise in the seroprevalence of the respective age-cohorts. The results of a recent study in Irish dairy cattle suggest that there is a genetic component to BoHV-1 susceptibility, such that not all animals in a herd are at equal risk of seroconversion [23]. Also the level of shedding can vary depending on the strain of the virus which may potentially explain some of the variation observed [3]. The introduction of vaccinated but uninfected animals into a non-vaccinating herd that tests for antibodies using a gB ELISA would also result in false positives and may add noisy deviations. We intentionally excluded herds with large numbers of introduced stock from the investigated data sets in order to minimise this issue. Nevertheless, small numbers of traded animals could still alter the apparent seroprevalence of an age-cohort. The introduction of serologically positive younger animals may add noise to the 0% cohorts, whereas introducing serologically negative older animals blurs the 100% in the respective cohorts. However, in practice that might not be a problem given that animal movements could be identified and excluded from defined age-cohorts until the profile category (red, blue, green in Figure 2) emerges. Introducing an animal undergoing primary infection on farm is compatible with either of the two scenarios in Figure 4 and “overwrites” the serological patterns from past virus circulation without the need for a reactivation event in the herd.

Both our survey data and model outputs present the ideal patterns of age-dependent seroprevalence profiles because trade of animals was excluded or suppressed. We are aware that in practice many herds will not be closed (i.e. through homebred replacement) with movements both into and out of the herd over several age-cohorts. As depicted in Figure 3A, this can result in noisier age-related seroprevalence profiles and may eventually blur the critical age cohort. However, modern cattle farming includes the recording of animal movements on the herd; thus, providing an effective correcting tool. The exclusion of introduced animals from the calculation of seroprevalence by age-cohort restored the predicted profile and the associated age-related threshold (Figure 3B).

Our understanding of the dynamics of BoHV-1 seroprevalence has been greatly improved by comparing aggregated serological results (across herds) with an interpretation of outcome patterns (age-dependent seroprevalence). Indeed, the benefit of visualising data in this more complex but complete manner has been recognised previously as a more appropriate starting point prior to technical aggregation [38]. In the current study, summarizing unmatched seroprevalence data across herds disregards the non-synchronised occurrence of BoHV-1 outbreaks in different herds, with the potential for a misleading interpretation of the resultant patterns. We have demonstrated the utility of explicitly visualising data of age-related serological surveys according to individual herd profiles. This approach moves the focus from an observation of an apparent trend across several age-cohorts towards one immediately centred on the youngest age cohort with seroprevalence close to 100%. Indeed, identification of the youngest age cohort involved in the last outbreak already indicated that serological profiles of any of the older age-cohorts in the herd will tend toward 100% seropositivity.

In Irish dairy farming, different management groups (e.g. youngstock, lactating cows) often form independent epidemiological units, due to, for example, separate housing or grazing on separate blocks of land. There would be a need for an independent evaluation of seroprevalence profiles within each management group, if these were found to be epidemiologically distinct on a single farm. Since we were not able to extract these management groups from the available data, we considered a whole herd as a single epidemiological unit in our modelling work.

By coupling the outcome of our data analysis with simulation output of an individual-based model at the herd scale, we have shown that the youngest age-cohort with a high seroprevalence profile in a naïve herd is related to the time interval since infection ceased to circulate (while still being present in latent form in seropositive animals). It is intrinsic to the interpretation of age-dependent seroprevalence profiles that only the most recent outbreak can be deduced from the profile. Indeed, a successful reactivation event (i.e. one that initiates further primary infections) within the herd or the introduction of the virus by other pathways (that results in active virus circulation) will each result in a further outbreak that will mask the previous serological profile (e.g. Figure 6).

### Effect of vaccination

The main benefit of the age-threshold approach comes with longitudinal surveys tracking the progression of the youngest age-cohort with a seroprevalence close to 100%. Particularly in relation to vaccination, this type of longitudinal view may demonstrate to stakeholders the efficacy of the intervention (Figure 5). In the case herd in this study, the data did not reveal any virus circulation after the herd started vaccination. This is plausible according to experimental and field data from several studies which demonstrated that different BoHV-1 vaccines can effectively reduce the severity of clinical signs, the shedding of infectious material and, most beneficially, the frequency of reactivation [8, 11, 37, 39]. Replacement of old seropositive stock by seronegative home-bred young-stock led to a continual shift of the age-threshold of the seroprevalence profile towards older animals confirming the absence of another major episode of virus circulation since vaccination was commenced. This pattern was observed also in other vaccinating herds with minimal in-moves (see Additional file 3).

Intrinsic to the interpretation of age-dependent seroprevalence profiles (see Figure 5), it is not possible to conclude whether there has been more than one major outbreak in a herd or what the timing was between these outbreaks. Logically therefore, an equivalent shift in the age-threshold in the seroprevalence profile would appear if reactivation events or external introduction of virus (for example through contamination of people or equipment, or buying-in of animals with primary infections) had not occurred since 2011, irrespective of whether the herd had been vaccinated or not (see Figure 2). Therefore, we cannot directly attribute the dynamics in Figure 5 to the effectiveness of the vaccine. An identified exposure to BoHV-1 subsequent to the introduction of vaccination is needed in order to demonstrate that vaccination was protecting this herd from further major outbreaks. A single seroconversion due to field infection in age-cohorts younger than the age-threshold would provide evidence of herd challenge and protective effect of herd vaccination.

Nevertheless, the longitudinal pattern shown in Figure 5 gives a picture of how the effect of vaccination on the epidemiology of age-related BoHV-1 prevalence can be investigated in future research and surveillance. Consistent vaccination is cited as the main factor in reducing prevalence in countries with high seroprevalence and is therefore suggested as an essential tool at the start of eradication programmes [1].

### Implications for herd testing and virus control

We have elaborated how age-related seroprevalence improves the understanding of output-related patterns of serological data regarding BoHV-1 infection. Given our intention to provide decision support for the design and implementation of effective control measures, this improved understanding can be translated to recommendations to make the best use of testing and vaccination. Age-related serological data, as used at individual herd level in our study, can indicate whether BoHV-1 infection was recently circulating in a herd (red in Figure 2) or only in the past (blue in Figure 2). The identified categorisation supports the interpretation of the BoHV-1 related status of a herd and is more informative than considering changes in the overall herd seroprevalence level.

The conceptual understanding of a critical age-threshold per individual herd in a serological survey translates into alternative testing strategies to address different questions (see Table 1). First, to inform a screening survey targeting the presence of BoHV-1 in a population, the most reliable source are older age-cohorts. Second, the most informative source for serological surveillance of recent BoHV-1 circulation would be the youngest age-class in a herd that excludes interference with maternal immunity and has epidemiological contact with older latently infected animals. Third, in order to monitor efficiency of vaccination and associated control measures, the annual increase in the age-threshold of the seroprevalence profile would be supported by surveillance of potential new exposure via the testing in younger age-cohorts. For dairy herds, screening of first lactation animals would be a useful tool to confirm that BoHV-1 did not continue to circulate.

**Table 1:** **Derived implications for herd testing and surveillance.**

Surveillance objective	Proposed approach
Detection of BoHV-1 antibodies in a population	Concentrate test capacity on older age-cohorts
Detection of recent BoHV-1 circulation in a population	Concentrate test capacity on the youngest age-cohort without maternal immunity but with epidemiological contact with older latently infected animals
Monitor efficiency of vaccination and other control measures	Observe the annual increase in the age of the age-threshold cohort based on the seroprevalence profile

## Conclusion

We confirm an age-related trend in the prevalence of BoHV-1 antibodies. On average, older animals are more often seropositive than youngstock. However, analysis of individual herd profiles has demonstrated that major BoHV-1 outbreaks in dairy cattle herds affect animals independent of age and lead to almost 100% seroconversion in all age groups, or at least in all animals within a single epidemiological unit. Herds were identified whose seroprevalence profiles showed steep jumps between consecutive age cohorts, from a low to very high percentage of seropositive animals. The age-cohort in which this steep jump occurs will shift with every year without a new major outbreak and provide information on the BoHV-1 status and history of the herd. Therefore, it is useful to evaluate age-related data from serological surveys to identify the age-threshold cohort (the youngest age cohort with seroprevalence close to 100%) in each herd. This age-threshold cohort provides useful information for surveillance and monitoring. Population-based screening of BoHV-1 herd-level prevalence should target the older age-cohorts, whereas detection of recent virus circulation (i.e. major outbreaks) should address the youngest age-cohorts in a herd without maternal immunity but with epidemiological contact with older latently infected animals. Efficacy of vaccination or other intervention strategies (e.g. biosecurity) should be monitored by observing the longitudinal shift in the age of the age-threshold cohort and through surveillance of the younger cohorts to identify potential new exposure. Further research is needed to explore data for seasonal calving beef herds to see if patterns there are as clear as in dairy herds.

## Supplementary information

**Additional file 1: ODD protocol of the simulation model.**

**Additional file 2: Age-related prevalence profiles without seasonal calving.**

**Additional file 3: Effect of vaccination.**

## Data Availability

The dataset used in this study is the private property of farmers located in the Republic of Ireland. The data was made available for research purposes and cannot be made publicity available. The code of the model is available on request.

## Abbreviations

AIM: animal Identification and Movement
BoHV-1: bovine herpesvirus type-1
DAFM: Department of Agriculture, Food and the Marine
EU: European Union
ICBF: Irish Cattle Breeding Federation
IBR: infectious bovine rhinotracheitis
SD: standard deviation
