# Supplementary material for: Epidemiology of age-dependent prevalence of Bovine Herpes Virus Type 1 (BoHV-1) in dairy herds with and without vaccination
Source: Vet Res. 2020 Sep 25;51:124. doi: 10.1186/s13567-020-00842-5 (PMC7520977; doi:10.1186/s13567-020-00842-5)
Supplement: Supplementary file 1 — Additional file 1: ODD protocol of the simulation model. [file 13567_2020_842_MOESM1_ESM.docx]

Additional file 1: ODD protocol of the simulation model

## Overview

The within-herd simulation model is stochastic and individual-based. It simulates the spread of BoHV-1 in an Irish dairy herd. The model is documented according to the ODD (Overview, Design, Details) protocol [1, 2]. The model used here is a further development of previous modelling wok on Irish cattle herds [3, 4]. Adaptations were made to represent details of BoHV-1 epidemiology in typical Irish cattle farming.

The purpose of the model application is to understand how the age-dependent patterns observed in the BoHV-1 seroprevalence survey data from Irish cattle herds is influenced by non-synchronized epidemiological stages between herds at the time of data collection.

### Entities, State Variables & Scales

The model consists of two entities, namely the animal and the herd entity (see Additional figure 1). Each entity is described by one or a set of components. Components in turn are groups of state variables (listed in green rectangles) that describe the entity. These components can be added to an entity or removed, depending on whether they are currently used or not. In the following description of the model, component names are marked in blue font, whereas state variables are indicated in green.

In the model, individual animals are the most complex entities. Each individual animal comprises at least an **AnimalBase** component that provides information on typical demographic variables such as an animal’s age, sex, breed, dateOfDeath or membership in a managementGroup. In addition, each animal holds an **EpiStatus** component that keeps information on the animal’s BoHV-1 infection state and when it last changed. Female animals that have reached a specific age or are intended for breeding get the **AwaitsBreeding** component attached. This component stores all state variables necessary for breeding, such as the number of inseminationTrials, the lastInseminationTrial, whether an insemination was successful (inseminatinoSuccess) and, if so, the date of conception (dateConception).


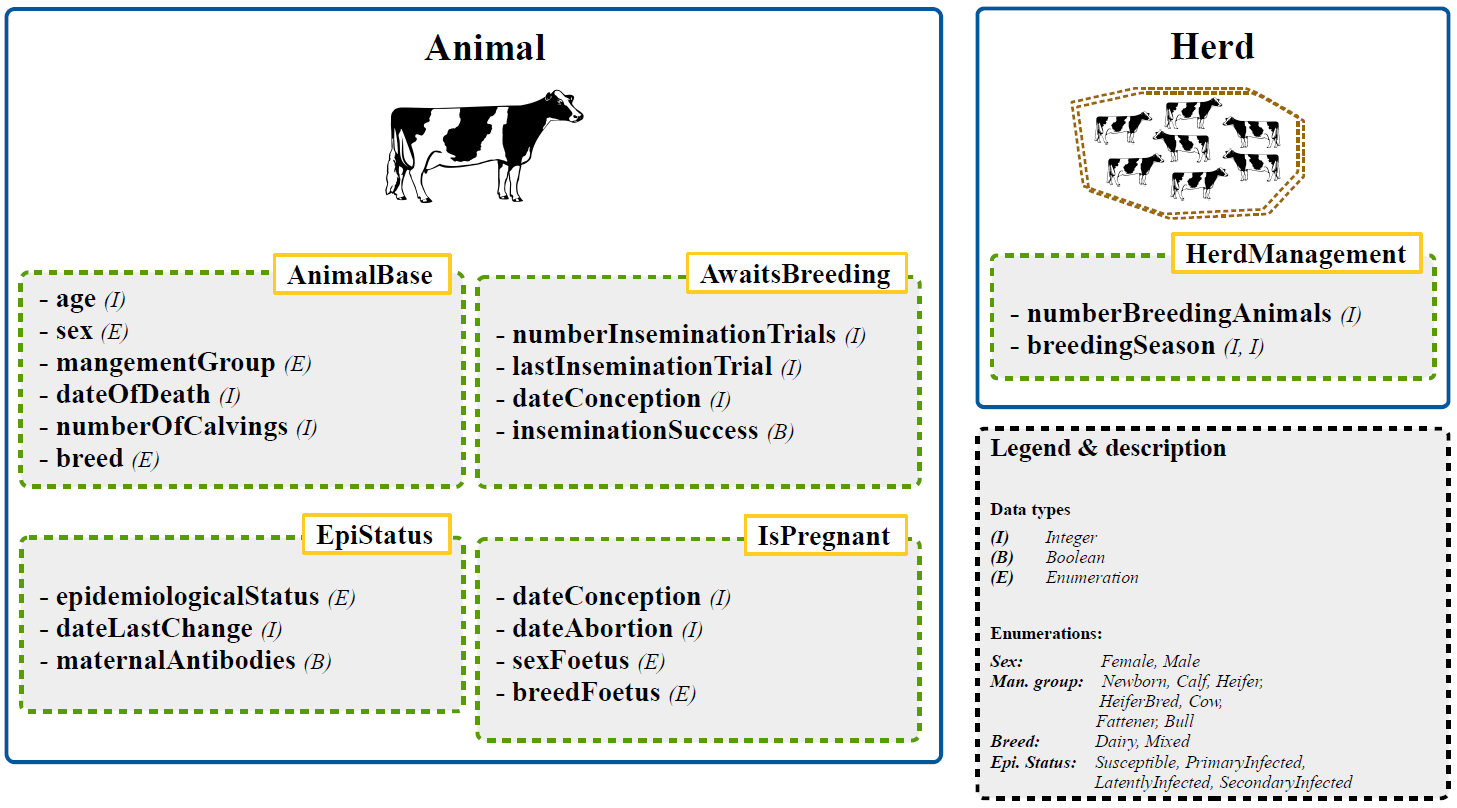


Additional figureS1: Overview of the model entities (blue rectangles) and components (green rectangles) with its associated state variables.

### Process Overview & Scheduling

The model runs in discrete time steps of one week. The main processes are executed in the following order:

1. Grouping
2. Breeding
3. Calving
4. Abortion
5. Sale decision
6. Mortality
7. BoHV-1 infection

## Design Concepts

Basic principles:

Animals reproduce according to biological parameters, while breeding is managed through the herd. The epidemiological states of animals are managed by an infection module. The herd replacement rate is derived by the predetermined stocking size (number of breeding animals).

Emergence:

The demographic structure of the herd emerges from biological life-cycle parameters of individual animals. Disease outbreaks emerge from individual disease courses triggered by animal-to-animal transmission.

Stochasticity:

Most processes and decisions in the model are stochastic.

## Details

### Initialization

The age-structure of the simulated herd is initialized by a randomly drawn representative from a preparatory simulation (40 years) of cattle management. This ensures a proper initial herd structure and valid animal components and state variable values. After initialization and at the start of the final simulation each animal in the herd is seronegative.

### Input data

Except for the table used for initialization, the model does not use input data.

### Sub-models

In accordance with [5], description of sub-models is arranged into biological-related, farming-related and pathogen-related processes of the disease model.

#### Biological-related processes

Mortality:

The model accounts for a background mortality, representing the constant weekly risk that an individual animal will die a natural death. Instead of evaluating mortality at each simulation step, life expectancy is calculated for each animal (whenever an animal is added to the system/model) based on the annual background mortality parameter *m*

$$LifeExpectancy= - \left( \frac{log(x)}{m | m_{elderly}} \right)$$

with *x* being a random number between 0 and 1 *{x ε ℝ | 0 ≤ x ≤ 1}*. If the calculated LifeExpectation exceeds a predefined threshold (*LifeExpectancyThreshold*) a new life expectancy is calculated by a means of an increased mortality (*m_elderly_*). The dateOfDeath is calculated accordingly and set in the **AnimalBase** component.

Ageing:

Each individual in the model is subject to an ageing process that increases the state variable age of each animal by one week per simulation step. Ageing can trigger other processes, for example the transition between management cohorts.

Pregnancy & calving:

If breeding (see submodel breeding) is successful, animals are converted to pregnant. The **AwaitsBreeding** component is detached and animals get the **IsPregnant** component. The state variable dateOfConception is retained from the **AwaitsBreeding** component to calculate the time of calving. The sex of the fetus (sexFoetus) is determined stochastically according to $P_{female}$. The fetus’ breed (breedFoetus) is set to mixed with probability P_mixed_.

Pregnant animals calve 285 days (durationPregnancy) after successful conception. At calving the state variables sexFoetus and breedFoetus are retained from the **isPregnant** component and the newborn animal gets this information assigned to its **AnimalBase** component. After calving the **IsPregnant** component is detached from the dam and the **AwaitsBreeding** component is attached. If a heiferBred has calved it will convert to the cow cohort.

The BoHV-1 epidemiologicalStatus variable from the **EpiStatus** component of the newborn calf is set to susceptible but depending on the epidemiologicalStatus of the dam the state variable maternalAntibodies is set to true.

Abortion:

Pregnant heifers or cows abort their fetus stochastically. The decision whether and when a pregnant animal aborts is made after positive conception. First, we generated a random number to decide whether an animal had aborted, based on the overall probability of abortion from all conceptions. Then, if abortion is calculated to have occurred, the state variable dateAbortion from the **IsPregnant** component is determined by drawing a random number between 0 and 285 (gestationLength)

#### Farming-related processes

Grouping:

Each animal in the model is assigned to one of the following managementGroups: Newborn, Calf, Heifer, HeiferBred, Cow & Fattener. Information on the managementGroup is stored in the **AnimalBase** component.

When an animal of managementGroup Newborn has reached a certain age (maxAgeNewborn) it will convert to managementGroup Heifer, if its sex is female and its breed is dairy. Male animals are sold two weeks after birth (ageSellMaleCalf). Heifers can become HeiferBreds and cows, subsequently. Cows can become fatteners. This is handled in the breeding and calving component.

Breeding:

Cows and Heifers eligible for breeding are inseminated when the herd is in its breedingSeason (see **HerdManagement** component). Animals are eligible for breeding if breeding success has not yet been determined and they have exceeded the waiting time (waitingTimeBreeding) after their last calving. Heifers are eligible for breeding at minimum age (minAgeBreeding). All eligible animals are inseminated and it is decided stochastically by drawing from a predetermined distribution (distributionConception) whether an animal will be successful inseminated (inseminationSuccess) and when successful bred animals will conceive (dateConception).

Animals that were successfully inseminated are converted to pregnant when the model step equals their dateConception. Component **AwaitsBreeding** is detached and the **IsPregannt** component is attached.

If the animal is in the Heifer cohort, its managementGroup is changed to HeiferBred after successful breeding. Animals without breeding success are transferred to the Fattener cohort.

#### Pathogen-related processes

Disease course:

In the model, each individual animal is assigned to one of four BoHV-1 epidemiologicalStates (see Additional figure 2). Healthy animals that have never been exposed to BoHV-1 are susceptible animals. If a susceptible animal becomes infected it enters the primary infection state and will be a lifelong carrier of the virus. The infectious period of a primary infected animal is two weeks. During this period the animal sheds high levels of virus and is highly contagious. After primary infection hosts recover and enter the latent infection health-state. Latently infected animals are still BoHV-1 positive but do not shed the virus. In reality, latently infected animals can reactivate the virus, which causes a secondary infection. However, in this modelling approach the event of reactivation was not modelled explicitly and latently infected animals remain so for the rest of their lives, unless explicitly modelled.


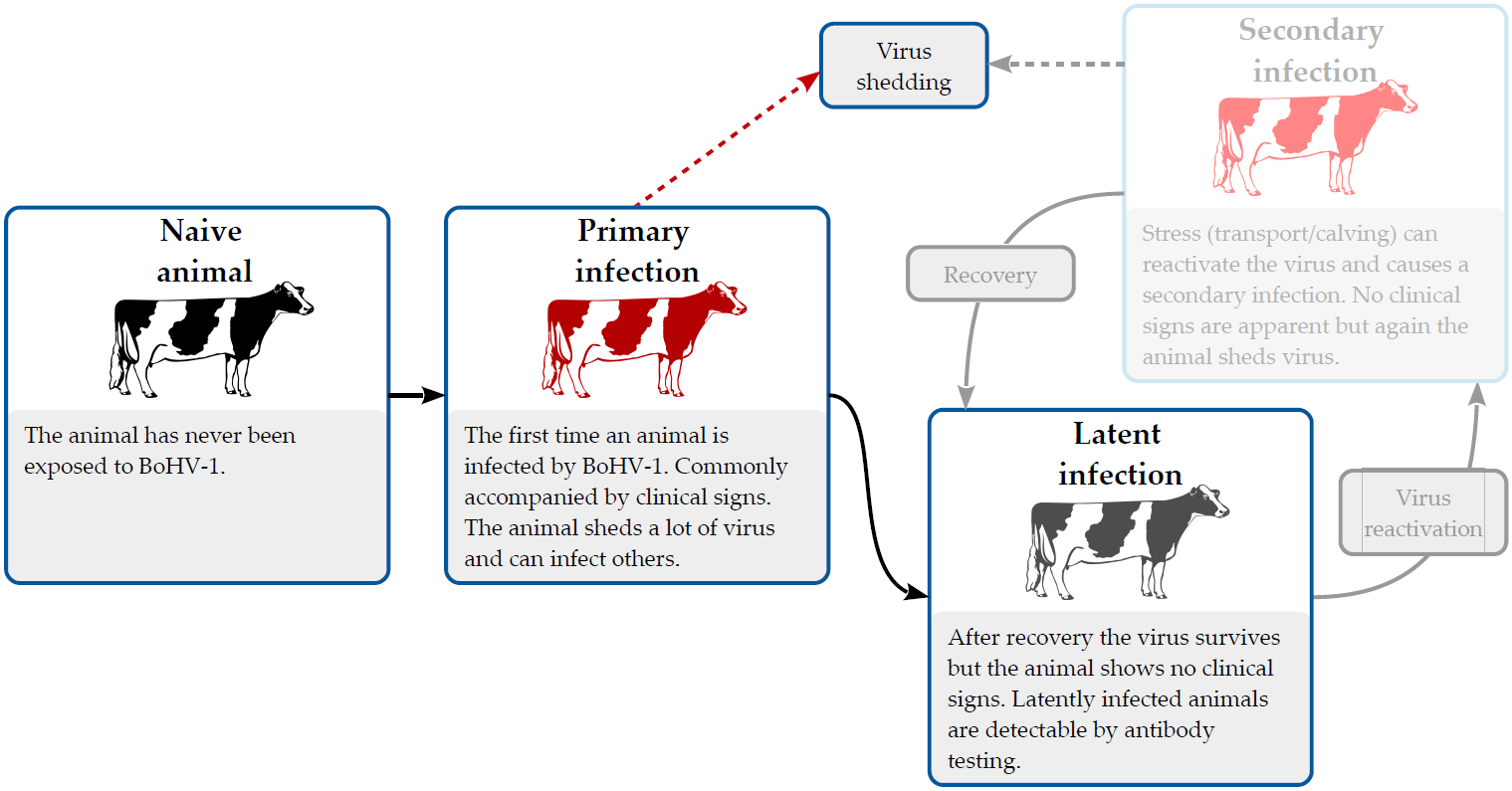


Additional figure S2: BoHV-1 health-states and infection cycle.

Virus transmission:

The event of getting infected with BoHV-1 is modelled stochastically by means of a frequency-dependent transmission rate that is converted into an individual probability of getting infected. The frequency-dependent transmission rate accounts for the event of contracting the infection via direct contact (R_DT_). At each simulation step the rate is recalculated and converted into an individual weekly probability of getting infected (P_inf_):

$$P_{inf}=1-{exp}^{\left( -R_{DT} \right)}$$

The way of calculating the respective rate is explained in the following. In the model, animals in a herd are split into separate management pools (*p*). For the dairy herd modelled here, two management groups are accounted for, namely youngstock (female calves, heifers) and the remaining stock (e.g. cows). As stated before, a reactivation event was not modelled explicitly. For each susceptible animal in management group *p* the rate of virus transmission as a result of infection through direct contact is calculated by means of the following model:

$$R_{DT}=\beta_{PI}\frac{{PI}^{\left( p \right)}}{N^{\left( p \right)}}+\left( \beta_{PI}^{b}\frac{\sum_{c´\neq c} {PI}^{\left( P^{'} \right)}}{\sum_{c´\neq c} N^{\left( P^{'} \right)}} \right)$$

with ${PI}^{\left( p \right)}$ being the number of primary infected animals in management group *p*; $N^{\left( p \right)}$ being the number of animals in management group *p* and $\beta_{PI}$ the transmission coefficient per week, associated with the primary infected animals. Infection between management groups was also accounted for in the model. A second transmission coefficient for virus transmission between management groups $\left( \beta_{PI}^{b} \right)$ was calculated.

## Parameters

| Parameter | Symbol | Type | Value | Description |
| --- | --- | --- | --- | --- |
| gestationLength |  | int | 40 w (285 d) | Length of gestation/pregnancy in weeks |
| maxAgeNewborn |  | int | 2 w | Age to move from Cohort Newborn to Calf |
| maxAgeCalf |  | int | 26 w (6 m) | Age to move from Cohort Calf to Heifer |
| maxAgeFattener |  | int | 160 w (≈3 y) | Maximum age for Cohort Fattener |
| minAgeBreeding |  | int | 61 w (14 m) | Minimum age of heifers for first breeding |
| ageSellMaleCalf |  | int | 2 w | Age to sell male dairy calves |
| ageSellMixedCalf |  | int | 2 w | Age to sell mixed breed dairy calves |
| probFertility |  | float | 0.95 | Fertility of animals in dairy herds |
| waitingTimeBreeding |  | float | 8 w | Waiting time between calving and breeding attempt |
| distributionConception |  | List[float] | [0.04 0.14 0.16 0.14  0.12 0.1 0.08 0.07  0.05 0.04 0.03 0.03] | Distribution of conception time |
| distributionBinSize |  | int | 3 w | Bin size of distributions in weeks |
| probFemale |  | float | 0.5 | Sex ratio: share of female calves |
| abortionProb |  | float | 0.03 | Probability of abortion |
| betaPiWithin |  | float | 1.0 | Beta for primary infected animals within pools (calibrated based on a R0 of 3.5) |
| betaPiBetweenPools |  | float | 1.0 | Beta for primary infected animals between pools |
| stepsInfected |  | int | 2 w | Duration of infection in weeks/model steps |
| durationMAB |  | int | 26 w (6 m) | Duration of presence of maternal antibodies in weeks/model steps |
| backgroundMortality |  | float | 0.03 | Annual background mortality |
| backgroundMortalityNewborn |  | float | 0.87 | Annual background mortality for cohort Newborn |
| backgroundMortalityCalf |  | float | 0.05 | Annual background mortality for cohort Calf |
| elderlyMortality |  | float | 0.4 | Annual background mortality for old animals |
| elderlyThreshold |  | int | 182 w (3.5 y) | Age threshold for old animals |
| FattenerMortality |  | float | 3.0 | Annual background mortality for animals in the Fattener cohort |
