# Supplementary material for: Epidemiology of age-dependent prevalence of Bovine Herpes Virus Type 1 (BoHV-1) in dairy herds with and without vaccination
Source: Vet Res. 2020 Sep 25;51:124. doi: 10.1186/s13567-020-00842-5 (PMC7520977; doi:10.1186/s13567-020-00842-5)
Supplement: Supplementary file 2 — Additional file 2: Age-related prevalence profiles without seasonal calving. [file 13567_2020_842_MOESM2_ESM.docx]

Additional file 2: Age-related prevalence profiles without seasonal calving


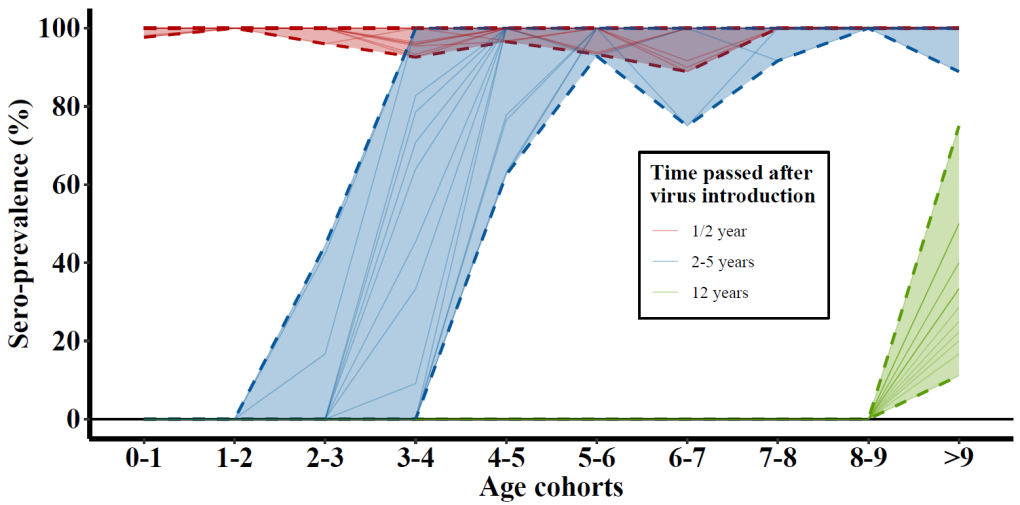


Model output of the age-dependent seroprevalence profiles of 15 simulated cattle herds at three subsequent time points after a single introduction of BoHV-1 infection (line colours). In this scenario all year round calving was modeled.
