# Supplementary material for: Epidemiology of age-dependent prevalence of Bovine Herpes Virus Type 1 (BoHV-1) in dairy herds with and without vaccination
Source: Vet Res. 2020 Sep 25;51:124. doi: 10.1186/s13567-020-00842-5 (PMC7520977; doi:10.1186/s13567-020-00842-5)
Supplement: Supplementary file 3 — Additional file 3: Effect of vaccination. [file 13567_2020_842_MOESM3_ESM.docx]

#

Additional file 3: Effect of vaccination

We divided the herds with multiple tests (Dataset B) into two investigation groups to study the effect of vaccination on within-herd prevalence (Additional figure 4). The first group consisted of six vaccinated herds (blue lines) that were already vaccinating before testing was conducted (solid blue lines) or started to vaccinate vaccination (dotted grey line) within the sampling period (Additional figure 4a). Eleven non-vaccinated herds (red lines) served as a control group (Additional figure 4b). In all vaccinated herds, seroprevalence declined during the period in which the herds were surveyed. Different patterns became visible in the non-vaccinating herds (Additional figure 4c). While for most of the herds seroprevalence remained constantly at a very low level, two major outbreaks occurred.


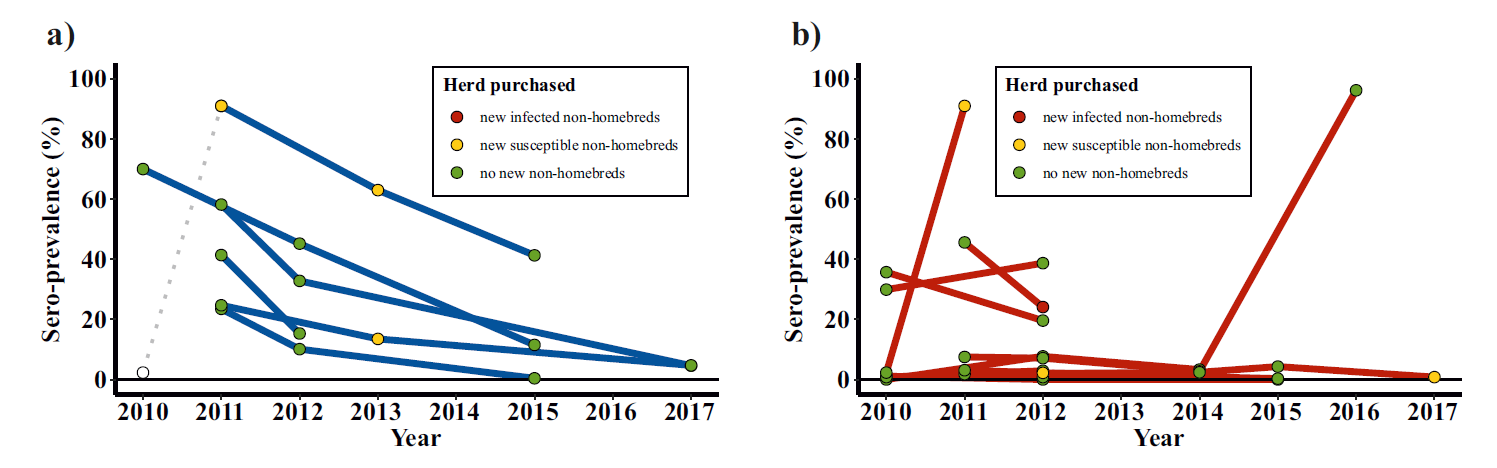


Additional figure S4: Effect of vaccination on the within-herd seroprevalence. In the plot each line represents one multiple tested herd along with the year in which the herd was tested. (a) Effect of vaccination on the temporal within-herd seroprevalence in vaccinated dairy herds. For each herd the test year is indicated by coloured dots, providing information on whether the herds contained any non-homebred animals at the point of sampling. (b) Temporal development of within-herd seroprevalence in non-vaccinating dairy herds.


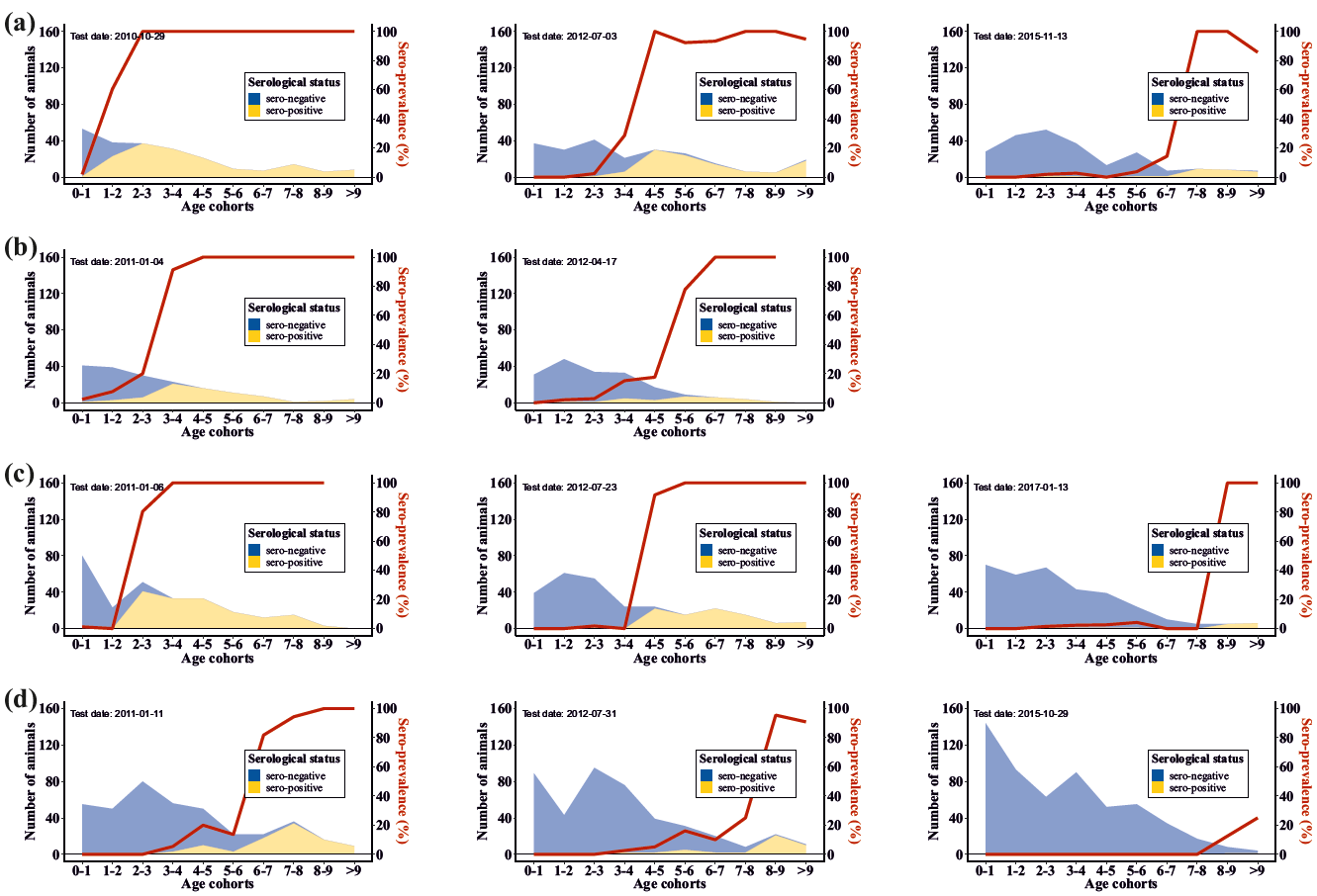


Additional figureS 5: Effect of vaccination on the age-related seroprevalence in single herds (a-d) at multiple time points.
